# Acute interstitial nephritis due to flecainide therapy in the 38^th^ week of pregnancy

**DOI:** 10.1186/s12882-016-0240-8

**Published:** 2016-03-15

**Authors:** Julius J. Schmidt, Leyla Ramazan, Clemens Bockemeyer, Hans-Heinrich Günter, Jens Martens-Lobenhoffer, Tina Ganzenmüller, Stefanie M. Bode-Böger, Jan T. Kielstein

**Affiliations:** Department of Nephrology and Hypertension Medical School Hannover, Carl-Neuberg-Strasse 1, 30625 Hannover, Germany; Institute of Pathology, Medical School Hannover, Hannover, Germany; Department of Gynecology and Obstetrics, Medical School Hannover, Hannover, Germany; Institute for Clinical Pharmacology, Otto-von-Guericke University, Magdeburg, Germany; Institute of Virology, Medical School Hannover, Hannover, Germany; Medical Clinic V, Academic Teaching Hospital Braunschweig, Braunschweig, Germany

## Abstract

**Background:**

Acute interstitial nephritis (AIN) represents a frequent cause of acute kidney injury. While many etiologies of AIN have been recognized, the majority (60–70 %) are due to allergic reactions or drug exposure. Many different classes of drugs and several agents within a class can cause drug induced AIN. Flecainide, a class Ic antiarrhythmic drug, had thus far not been associated with the occurrence of AIN.

**Case presentation:**

Here we describe a case of biopsy proven AIN after flecainide therapy in a pregnant patient. The 24-year old Caucasian woman was admitted to our university hospital for a planned c-section. She had been put on flecainide at a dose of 200 mg/d for supraventricular tachyarrhythmia of the fetus ten days earlier. The only fleaainide drug level was obtained 24 h after the last dose. At this time point the serum level was still in the therapeutic range (392 ng/mL). After hospital admission the patient underwent uneventful c-section and delivered a 3095 g baby girl with mild insufficiency of the tricuspid valve. In the hours following the c-section, a single dose of the non-steroidal anti-inflammatory drug (NSAID) ibuprofen (600 mg) as well as single dose of diclofenac (100 mg) was administered. Within 5 days after c-section her baseline creatinine of 30 μmol/L increased to 277 μmol/L. The serum creatinine continued to rise to 411 μmol/L on hospital day # 7. On renal ultrasound kidneys were enlarged and swollen. Urinary sediment at this point only revealed slight proteinuria (506 mg/g creatinine). A renal biopsy was performed showing acute interstitial nephritis. Within four days the renal function improved after discontinuation of flecainide and NSAIDs even without steroid therapy and the patient was discharged with a creatinine of 88 μmol/L after 13 days in the hospital.

**Conclusion:**

This case suggests that flecainide, at least in combination with NSAIDs, can cause AIN.

## Background

Acute interstitial nephritis (AIN) represents a frequent cause of acute kidney injury. While many etiologies of AIN have been recognized, the majority (60–70 %) are due to allergic reactions or drug exposure [1]. Many different classes of drugs can cause AIN ranging from more frequent ones like antimicrobials (ampicillin, ciprofloxacin, methicillin, rifampicin and sulfonamides) and non-steroidal anti-inflammatory drugs (acetylsalicylic acid, ibuprofen, naproxen) over proton pump inhibitors (omeprazole and pantoprazole) [1] to rarer causes like newer quinolones (moxifloxacin) [2] and/or iron chelating agents such as deferasirox [3]. Flecainide, a class Ic antiarrhythmic drug, had thus far not been associated with the occurrence of AIN. It is used to treat a variety of cardiac arrhythmias including paroxysmal atrial fibrillation, paroxysmal supraventricular tachycardia and ventricular tachycardia. During pregnancy, flecainide is used to treat refractory fetal tachycardia. It blocks sodium channels in the heart, causing prolongation of the cardiac action potential. The majority of flecainide is eliminated by the kidneys, with the remainder metabolized by the cytochrome P450 2D6 isoenzyme in the liver [4]. Despite the narrow therapeutic index of flecainide reflected by the fact that the toxic effects of flecainide are closely related to the plasma levels of the drug [5], therapeutic drug monitoring is not regularly performed.

## Case presentation

**Fig. 1:**
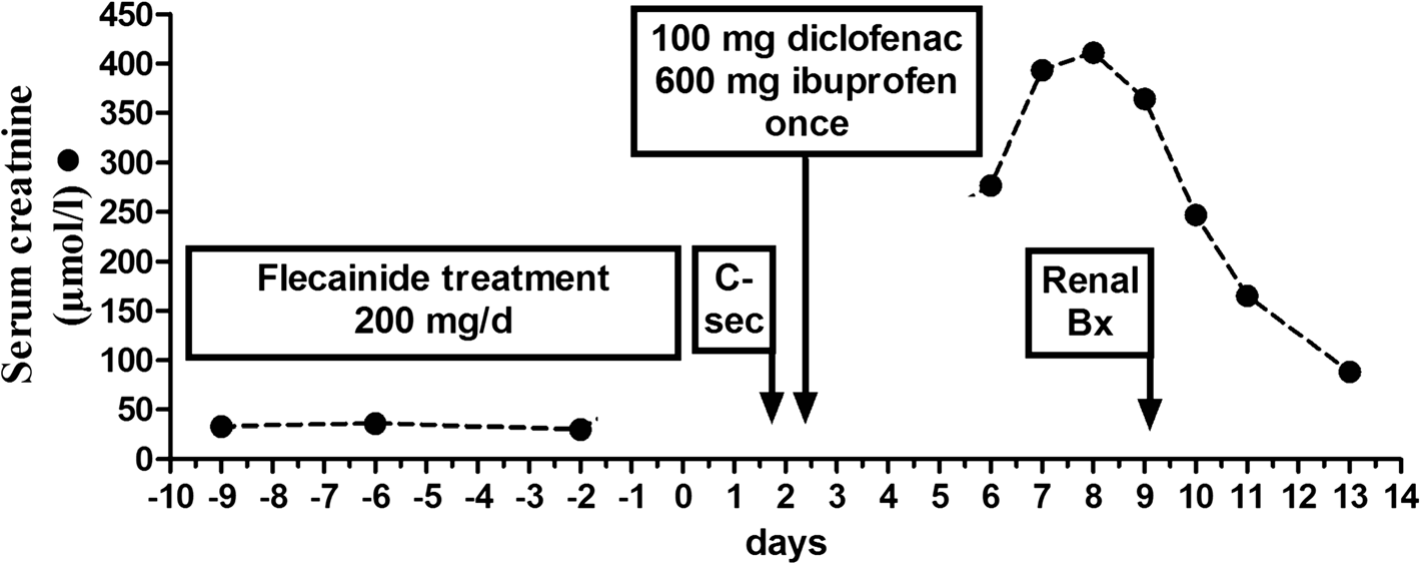
Serum creatinine CRP levels of a pregnant patient during outpatient visits as well as during the hospital course/treatment of AIN due to flecainide (and NSAID). c-sec = c-section. Renal Bx = renal biopsy

**Fig. 2:**
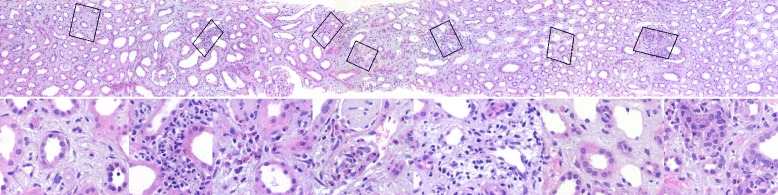
Kidney biopsy (HE Stain, 100 X)

Here we describe a case of biopsy proven AIN after a 10 day flecainide therapy and a single dose NSAIDs in a pregnant patient. Due to fetal tachycardia the 24-year old woman (height 162 cm, weight 86.7 kg) was put on flecainide therapy in the 36^th^ week of gestation (G2P0). Ten days later she was admitted to our university hospital for a planned c-section due to fetal heart failure. On admission she reported no complaints. The blood pressure was 109/72 mmHg and the heart rate was 108 beats per minute. The remainder of the physical exam was unremarkable. She denied any regular medication other than vitamin supplementation. Laboratory evaluation showed a slightly increased alkaline phosphatase of 134 U/L. The serum creatinine was 30 μmol/L. Her prior medical history was significant for two events of peripheral self-limiting facial nerve palsy. In the cardiotocography the fetal heart rate was 187 beats per minute. It also showed signs of slight uterus contractions. Fetal ultrasound revealed hypertrophic cardiomyopathy and tricuspid valve insufficiency grade two. One day after discontinuation of her 10 day flecainide therapy, her serum level was 391 ng/mL (therapeutic range 300–1000 ng/mL) confirming adherence to the prescribed medication. At the day of delivery our patient received a single dose of the non-steroidal anti-inflammatory drug (NSAID) ibuprofen (600 mg) as well as single dose of diclofenac (100 mg). Within five days after delivery her baseline creatinine rose from 30 to 277 μmol/L. The same day treatment with cefotaxime was started for suspected urinary tract infection accompanied by a C-reactive protein (CRP) level of 68 mg/L. Despite the fall in inflammatory parameters serum creatinine continued to rise up to 411 μmol/L on hospital day # 7 (Fig. 1). On renal ultrasound, kidneys were enlarged and swollen (323 ml for the right and 326 ml for the left kidney). There were no signs of post-renal AKI. Urinalysis showed proteinuria, hemoglobinuria and leukocyte esterase. Urinary sediment at this point only revealed slight proteinuria (506 mg/g creatinine) and white blood cells (28/μl). Red blood cells or urinary casts were not detectable. Renal biopsy was performed on hospital day # 10 (nine days after delivery). Light microscopy (Fig. 2) revealed AIN. About 30 % of cortex and 10 % of medulla were infiltrated by a mixed infiltrate of lymphocytes, histiocytes, a few plasma cells and a maximum of 40 eosinophilic granulocytes. Beside this infiltrate there were also some scattered fibroblasts producing the matrix detected. However there were also many widened peritubular capillaries indicating a predominant AIN. Almost all tubules of the cortex were severely flattened and demonstrated a severe loss of the brush border, which highlighted a severe tubular damage. Furthermore focal areas of tubulitis of three intraepithelial lymphocytes per ten tubular epithelial cells (matching t1 according to Banff) were found. Within six days the renal function improved even without steroid therapy and the patient was discharged with a creatinine of 88 μmol/L after 13 days in the hospital (Fig. 1). Considering the low grade evidence supporting steroid treatment in AIN and given the spontaneous resolution of renal dysfunction with simple discontinuation of the offending agents, steroids were not deemed necessary.

## Discussion

Here we report to our knowledge the first case of biopsy proven AIN due to flecaininde. So far there is only one report on acute kidney injury in relation to flecainide overdose published. Meurin and co-workers describe a case of AKI after initiating therapy with NSAID as a co-medication of flecainide and an ACE-inhibitor [6]. In their report the 76 year old patient was on flecainide therapy (200 mg/d) for three years. Fifteen days after initiation of treatment with indomethacin (125 mg/d) their patient presented with a serum creatinine of 300 μmol/L. After discontinuation of the indomethacin and flecainide the patient’s creatinine decreased to 153 μmol/L within two weeks.

In contrast to the former study, in which no renal biopsy was performed, we did prove the diagnosis by renal biopsy. Moreover, we also performed a post hoc analysis of the flecainide level. About 24 h after the last dose of flecainide the serum level was 390 ng/mL, which is well within the therapeutic range of 300–1000 ng/mL. Given the fact that the half-life of flecainide is about 20 h (60–70 hours in patients with severe kidney damage), it is possible that the drug level during treatment was within the supratherapeutic range. The recommended daily dose of flecainide in the therapy of supraventricular arrhythmia is 300 mg/d at most and should be well monitored in patients with impaired kidney function. Our case suggests that flecainide, at least in combination with NSAIDs, can cause AIN due to its renal elimination and severe toxicity after overdosage [7]. Above and beyond the inherent limitations of a case report we cannot exclude that the single dose of two NSAIDs (600 mg ibuprofen and 100 mg diclofenac) administered on the day of delivery, caused the AIN alone. This holds especially true as we do not have daily creatinine levels. The fact that the patient only received a single (rather low) dose of NSAIDs but was on fleacainide therapy for 10 days with at drug levels well in the therapeutic range even 24 h after discontinuation, which we consider to be suggestive of an potential overdose, makes us lean towards a causative role for flecainide. It may well be that the combination of a single dose NSAIDS on top of a 10 day therapy with flecainide caused AIN.

## Conclusion

In terms of pharmacovigilance clinicians should be aware of the potential side effect of flecainide (in combination with NSAIDs) and monitor renal function regularly. An alternative approach would be to avoid NSAIDs in patients treated with flecainide altogether, especially since there are many alternatives available that are not associated with an increased risk for AIN.

### Consent

Written informed consent was obtained from the patient for publication of this Case report and any accompanying images. A copy of the written consent is available for review by the Editor of this journal.
